# Understanding emerging bioactive metabolites with putative roles in cancer biology

**DOI:** 10.3389/fonc.2022.1014748

**Published:** 2022-09-29

**Authors:** Olivier Philips, Mukhayyo Sultonova, Beau Blackmore, J. Patrick Murphy

**Affiliations:** Department of Biology, University of Prince Edward Island, Charlottetown, PE, Canada

**Keywords:** cancer metabolism, bioactive metabolite, post-translational modification, metabolite-protein interaction profiling, cancer therapeutics

## Abstract

Dysregulated metabolism in cancers is, by now, well established. Although metabolic adaptations provide cancers with the ability to synthesize the precursors required for rapid biosynthesis, some metabolites have direct functional, or bioactive, effects in human cells. Here we summarize recently identified metabolites that have bioactive roles either as post-translational modifications (PTMs) on proteins or in, yet unknown ways. We propose that these metabolites could play a bioactive role in promoting or inhibiting cancer cell phenotypes in a manner that is mostly unexplored. To study these potentially important bioactive roles, we discuss several novel metabolomic and proteomic approaches aimed at defining novel PTMs and metabolite-protein interactions. Understanding metabolite PTMs and protein interactors of bioactive metabolites may provide entirely new therapeutic targets for cancer.

## Introduction

The importance of altered metabolism in most cancers to meet the biosynthetic requirements of cell proliferation, angiogenesis, and immune evasion is well established (1). Beyond these anabolic and catabolic roles of metabolites in cancer, metabolites also function as regulators of gene expression and protein activity which affects cancer cell phenotypes. These include the classical oncometabolite, 2-hydroxyglutarate, (2-HG) (1) which is structurally similar to α-ketoglutarate (α-KG) and functions in part by inhibition of α-KG-dependent histone demethylases (2). This inhibition results in widespread changes to DNA methylation and gene expression as the main tumor-promoting mechanism of action of 2-HG. Although this mechanism is well established, many α-KG-independent effects have also been proposed in recent years (3). Along these lines, the roles of the 1-carbon pool and S-adenosyl-methionine (SAM) in providing methyl groups for DNA methylation and the requirement for NAD+ metabolism in activating the sirtuin family of transcriptional repressors are additional classical examples of direct links between metabolism and cancer biology. The identification of these functional metabolites has led to the development of small molecule inhibitors of the 2-HG-producing IDH1/IDH2 mutant proteins in gliomas (4) and acute myeloid leukemia (5) as well as a variety of sirtuin inhibitors (6). Based on these classic examples, we propose that efforts to understand the full repertoire of bioactive metabolites could lead to the development of exciting new treatment approaches for cancer and may even serve as diagnostic biomarkers in some cases (7).

Emerging data from the fields of neurobiology, immunology, exercise physiology, diabetes, and others have recently unveiled bioactive roles for several endogenous metabolites in human cells. Ultimately, these newly revealed bioactive functions of metabolites result in phenotypes with wide-ranging importance from exercise metabolism to cancer biology. Some of these metabolites enact their bioactive roles as, previously unappreciated, PTMs of proteins. Like other PTMs such as phosphorylation, acetylation, and ubiquitination, these PTMs could reside on proteins with roles in cancer biology. Yet other newly appreciated bioactive metabolites have unclear mechanisms of action. Both the proteins decorated by, and interacting with, bioactive metabolites could serve as new targetable modalities in cancer. Fortunately, new proteomic and metabolomic technologies enable mapping of novel PTMs as well as metabolite-protein interactions, providing a unique opportunity to develop bioactive metabolite-inspired therapies. Here, we briefly summarize the most recent developments in metabolite based PTMs and other emerging bioactive metabolites with unknown mechanisms of action. We also discuss emerging technologies that are enabling the field to identify new PTMs and study their interactions with proteins to elucidate new bioactive roles for metabolites.

## Metabolites as post-translational and epigenetic modifiers

The effects of PTMs such as phosphorylation, glycosylation, acetylation, ubiquitylation, and methylation are well established in cancer biology. Indeed, the most prominent cancer-causing mutations result in dysregulated phosphorylation such as those residing in PI3K and Rb (8). Recent studies involving high-resolution mass spectrometry experiments have expanded the number of known types of PTMs, all of which most likely regulate signaling and gene expression related to cancer proteins. Here, we highlight the most recent discoveries in metabolic-based PTMs.

### Lactylation

Lactate is the major end-product of glycolysis and is excreted by cultured cancer cells undergoing aerobic glycolysis (9), known as the classic “Warburg Effect”. However, lactate itself alters the transcription of genes involved in metabolic reprograming, regulation of cell cycle, and proliferation (10). Recently, the presence of lactate modification of lysine residues (lactylation) on several core histones has been reported (11). Furthermore, lactylation of lysines on histone H3 appears to be modulated by glucose uptake and lactate levels in the cell (11). Beyond glucose flux and mitochondrial inhibitors and hypoxia have also been observed to amplify lysine lactylation. More recent evidence of lactylation in aerobic glycolysis has also been observed during macrophage differentiation under the control of the B-cell adapter for PI3K (BCAP) (12) and in lung myofibroblasts (13). Although preliminary evidence suggests that the histone deacetylase p300 may serve as a histone lactylase, the precise control of histone lactylation will be an important topic of further study. Although these data potentially provide a direct functional link between glycolysis and gene expression whether lactylation of histones is required or is correlative with methylation is currently still unclear. Future work demonstrating the effects of mutations in key lactylated lysine residues on methylation will be required to establish this effect.

### Serotonylation

Although the tryptophan metabolite, serotonin is a classical neurotransmitter, it has also been proposed to have both cancer growth-promoting and anti-cancer roles (14). Due to these conflicting roles, understanding novel mechanisms of action for serotonin will be critical to investigate the putative role of serotonin in cancer. Like lactylation, serotonin has also been observed as a metabolite-based PTM on GTPases and vascular proteins, ultimately playing a role in vasoconstriction (15, 16). More recently, mass spectrometry experiments have shown evidence of post-translational modification of histones by serotonin (serotonylation). Serotonlylation was observed to occur on glutamine 5 of histone H3 trimethylated lysine 4 (H3K4me3)-marked nucleosomes resulting in the presence of detectable H3K4me3Q5ser in several mouse tissues and cell lines (17). Although the addition of serotonin has been shown to be performed by transglutaminase 2 (TGM2), how this process is fully regulated is still unclear (15). Ultimately, serotonylated histones could alter methylation status and cell fate and have significant effects on tumorigenesis and we propose should be an active area of further study.

### Succinylation

Succinate is a fundamental TCA cycle metabolite but, like 2-hydroxyglutarate, has also gained attention as an oncometabolite in cancers harbouring mutations in succinate dehydrogenase (SDH) enzymes (18). At least some of the oncometabolite function of succinate occurs *via* lysine succinylation, which was observed in *E.coli* several years ago (19, 20) and has more recently been mapped on 779 human proteins by mass spectrometry (21). SDH loss in a variety of cancers also results in hypersuccinylation of many metabolic proteins (22) which may also contribute to dysregulated metabolism in cancer. In recent years, the study of lysine succinylation biology has been active, revealing roles for succinylation of APP and tau in Alzheimer’s disease (23) and for regulating core histones in human cells (24, 25). Like lactylation and serotonylation, the proteins that succinylate and desuccinylate lysine residues and their phenotypic relationships with cancer are only beginning to be explored. For example, SIRT5, which also has roles in cancer (26) has been proposed as a desuccinylating enzyme for metabolic proteins (21). A recent study also showed that carnitine palmitoyl transferase 1A (CPT1A)-mediated succinylation increased human gastric cancer invasion through succinylation of S100A10 at lysine 47 (27). Furthermore, lysine succinylation may occur by a non-enzymatic chemical reaction, originating directly from succinyl-CoA. This suggests that the abundance of succinyl-CoA would be one of the main governing factors of lysine succinylation. For example, it has been shown that succinyl-CoA could non-enzymatically succinylate BSA and ovalbumin *in vitro* in a concentration-dependent manner, demonstrating that succinylation may at least partly depend on intracellular succinyl-CoA levels (28). Continued efforts to determine the full plethora of succinylated proteins and mechanisms by which they are regulated will be important since they have the potential to reveal entirely new therapeutic avenues for cancer biology.

### Others

Although we have attempted to describe the more recently-identified metabolite PTMs (**Table 1**), others have been under study over the last 10-15 years, including different forms of lysine acylation such as crotonylation (29, 30). Like several other metabolite-based PTMs, crotonylation has mainly been observed on histones and has been shown to be dysregulated in hepatocellular carcinoma (31). S-nitrosylaton, the addition of an NO group to cysteine residues, is another well-established functional PTM that has critical roles in the immune response and also has roles in cancer (32) but may be pro- or anti-tumorigenic, depending on concentration.

**Table 1:** Recent metabolite-based post-translational modifiers of proteins.

Post Translational Modifier	Proteins Modified	Proposed Writers	Study
Lactate	Histone H3	BCAP, HDACp300	(11–13)
Serotonin	Histone H3	TGM2	(15–17)
Succinate	APP/Tau	unknown	(23)
	Core Histones	unknown	(22, 25)
	Many Proteins	SIRT5	(21)
	S100A10	CPT1A	(27)

## Recently revealed functional metabolites with unknown mechanisms of action

In addition to bioactive metabolites that are PTMs, several emerging metabolites that have been shown to have phenotypic effects on cells may also have roles in cancer cell biology that appear independent of catabolism and anabolism, but for which the mechanisms are poorly described. These metabolic discoveries provide an opportunity to target proteins that elicit functional metabolite mechanism of action that could provide therapeutic treatment in cancer.

### Methylmalonic acid

**Figure 1:**
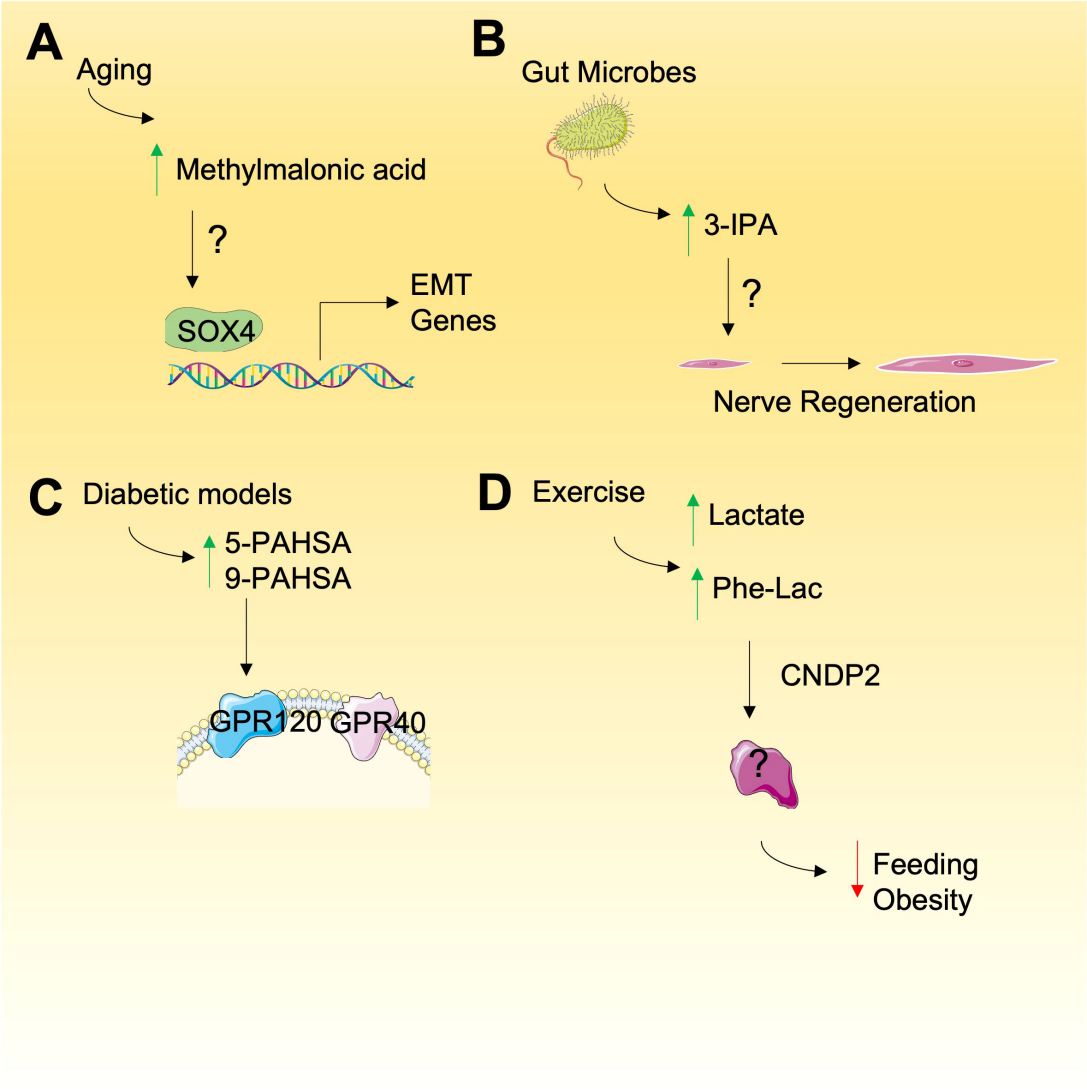
Emerging metabolites with unknown mode of action. **(A)** Methylmalonic is elevated during aging and may promote the expression of genes involved in epithelial-mesenchyme transition (EMT), relying on SOX4 but through undefined mechanism. **(B)** Gut microbes produce 3-IPA that result in nerve regeneration and other phenotypes by unknown mechanisms. **(C)** Increased PAHSAs have been observed in diabetic models that my have a role in cancer by activation of cell signaling through GPRs. **(D)** N-lactoyl-phenylalalanine (Phe-Lac) is elevated during exercise and functions in an unknown manner to reduce feeding and obesity in mice.

Methylmalonic acid (MMA) is a by-product of propionate metabolism that is best known as a marker for vitamin B12 deficiency due to its production by an impairment in L-methylmalonyl-CoA mutase (33). Recently, by applying both young and old patient serum to cultured cancer cells, MMA has been shown to be a key component of older individuals serum that promotes epithelial to mesenchymal transition and contributes tumor aggressiveness (34) (**Figure 1A**). Since cancer risk increases with age, the accumulation of MMA may link ageing and cancer progression and understanding MMA function may be a promising strategy to reveal new therapeutic targets for cancer. Mechanistically, the effects of MMA were shown to be dependent on TGFβ release which in turn induced SOX4 expression, a key promoter of EMT genes, in an autocrine manner (34). Prior work has shown that MMA caused CoEnzymeQ deficiency *in vitro (* 35), induced DNA damage in rat brain (36), impaired the differentiation of neuronal cells (37), and decreased cellular energetics in the C6 astrocyte-like cell line (37). Although together these data begin to explain the mechanism of action MMA, more research is required to determine the cellular targets of MMA and how it’s effects on EMT and tumor aggressiveness can be targeted therapeutically.

### Indole-3-propionic acid

Metabolites derived from gut microbiota are increasingly being shown to exert bioactive roles in cancer (38). Tryptophan-derived microbial products are of particular interest and in recent years several studies have revealed novel roles of 3-indolepropionic acid (3-IPA), a gut microbial-derived metabolite derived from tryptophan by *Clostridium sporogenes (* 39). More recently, 3-IPA has been shown to promote axonal nerve regeneration following intermittent fasting (40) (**Figure 1B**). Given the observations that intermittent fasting (IF) can have in providing anti-cancer benefits (41), the role of IF-induced 3-IPA should also be investigated. Indeed, 3-IPA has been shown to decrease cell proliferation in breast cancer (42) and protects against radiation toxicity (43). The metabolite may also suppress cancer immunity since recent untargeted metabolomic analyses revealed that 3-IPA is elevated in exhausted T cells and was shown to suppress T-cell responses when supplied exogenously (44). The effects of 3-IPA are proposed to be exerted through pregnane X receptor (PXR) and the aryl hydrocarbon receptor (AHR) (42, 43) but the full mechanisms of action remain to be elucidated.

### PAHSAs

About 10 years ago, intriguing, endogenously-produced, bioactive fatty acid esters of hydroxy–fatty acids (FAHSAs) were discovered in mice with proposed roles in regulating glucose levels (45). Of the hundreds of FAHSA isomers, the palmitic esters of hydroxy stearic acids (PAHSAs) have particularly strong bioactive roles as anti-inflammatory and anti-diabetic metabolites (46) and we propose their bioactive roles in cancer should also be investigated. Mechanistically, PAHSAs have been shown to activate G protein-coupled receptors known to be activated by free fatty acids. In this regard, both GPR120 (45) and GPR40 (47) are activated by 5-PAHSA and 9-PAHSA to regulate glucose uptake through glucose transporter 4 (GLUT4) translocation and glucagon-like peptide 1 (GLP-1) release (**Figure 1C**). Since GPR40 and GPR120 are expressed in cancers (48) and invoke specific signalling responses that lead to glucose uptake, PAHSAs may have bioactive roles in glucose uptake in cancer cells. Consistent with their function in glucose regulation, PAHSAs are also negatively correlated with high-fat diets and insulin resistance which may highlight an unexplored connection between PAHSAs, glucose uptake, cancer, and diet. However, although there may be a proposed positive link between PAHSAs and cancer through glucose uptake regulation, 9-PAHSA has been proposed to promote apoptosis in cells (49), others have not observed these effects in colon cancer cells HCT11-116 and HT-29 cells (50) suggesting the links between FAHSAs and cancer are complex. Ultimately, these findings emphasize the need for further study of FAHSAs in cancer biology.

### Phenyl-lactate

The avid production of lactate by cancer cells was originally made by Otto Warburg and is consistently observed in cancers and during bouts of intense exercise. Recently, N-lactoyl-amino acids, which are lactate-conjugated amino acids, have been detected by mass spectrometry and include N-lactoyl-isoleucine, N-lactoyl-tyrosine, N-lactoyl-tryptophan, and N-lactoyl-phenylalanine (51). It was recently shown that N-lactoyl-phenylalanine (Lac-Phe) is highly elevated during exercise alongside lactate and produced by carnosine dipeptidase 2 (CNDP2) in macrophages, monocytes and other immune and epithelial cells (52) (**Figure 1D**). By a, yet unknown mechanism, Lac-Phe has been shown to act as blood-borne signalling metabolite that suppresses feeding and obesity (52). Whether the concentrations of Lac-Phe used in an experimental setting reflect those found in circulation, either in healthy or cancer patients is not clear. Furthermore, the mechanism of action of Lac-Phe is completely unknown and investigating the role of Lac-Phe in cancer may provide insight into the bioactive role of Phe-Lac and reveal new targetable links between exercise-induced signaling metabolites and cancer.

### Others

These recently revealed bioactive metabolites whether acting as new post-translations on proteins or yet unknown functions, are not a comprehensive description of functional metabolites but a summary of more recent ones with potential roles in cancer. Others continue to emerge, whereby, for example, a recent untargeted mass spectrometry-based metabolomics study identified a form of 2-HG, 2-hydroxyglutarate-lactone, that exists in human cells for which the regulation and putative function apart from 2-HG is unknown (53). Although we do not summarize the vast number of diet-derived bioactive metabolites that also continue to emerge such as phytoestrogens (54). Furthermore, some endogenous metabolites act as riboswitches to influence gene expression (55). It is thus clear that although the metabolic “parts list” of metabolites in human cells is nearly complete, the next steps are to understand how they influence cancer phenotypes such as proliferation, metastasis, and immune evasion through their bioactive roles in cells.

## New tools to unveil metabolite PTMs and bioactive roles in cancer

Over the last few years, new mass spectrometry technologies have provided increasing opportunities to identify new bioactive metabolites or metabolite-based PTMs. Exploring chemical modifications on proteins, such as metabolites in an unbiased manner was first established using mass tolerant searching of LC-MS/MS spectra against predicted spectra based on the annotated proteome database (56). The speed and approachability of this strategy have improved with the release of tools like MSFragger (57, 58), TagGraph (59), MODa (60), and PIPI (61). These methods may also be deployed to search for novel metabolite-based PTMs in mass spectrometry datasets collected from bulk tumor tissue, cell lines, or histone extracts (62) under different conditions.

Understanding the bioactive roles of newly-discovered metabolites may best be achieved by determining protein-metabolite interactions (63) since they serve as conveyers of bioactivity in cells (64). Multiple groups have developed both protein-centric or metabolite-centric approaches to examine metabolite-protein interactions. The protein-centric approaches identify endogenous metabolites that potentially bind to a given, purified, protein target. Key among these approaches is Mass Spectrometry Integrated with equilibrium Dialysis for the discovery of Allostery Systematically (MIDAS) (65). This approach incubates purified proteins on one side of a metabolite-permeable dialysis membrane and examines differences in metabolite abundance on the opposite side by LC-MS. In preliminary findings, the approach has recently been deployed to identify metabolite interactions across 33 enzymes of central carbon metabolism (66) as well as with eukaryotic initiation factor 2B (eIF2B) (67), both of which reveal novel metabolite-protein interactions.

Alternative, metabolite-centric metabolite-protein interaction approaches aim to determine the protein interactors for a given metabolite and several of these approaches have emerged that can be divided into chemical probe-based and structure/stability-based (68, 69). The chemical probe-based approaches rely on metabolite derivatization with a tag that can be purified by affinity chromatography followed by target identification by mass spectrometry. This approach has mostly been deployed for identifying drug targets (70, 71). Probe-based approaches are challenging since the chemistry must be redesigned for each metabolite and may also affect the binding to the cognate protein. Alternative to probe-based approaches, stability-based strategies measure the differences in biophysical properties of proteins on a global scale imparted by unmodified small molecule drug or metabolite binding. Key among these approaches is thermal proteomic profiling (TPP), which monitors protein thermal stability over a temperature gradient by combining the principles of a non-global cellular thermal shift assay (CETSA) with multiplexed quantitative mass spectrometry‐based proteomics (72). TPP has been successfully applied to identify targets and off‐targets of drugs and to study the protein interactors of the crucial metabolite, ATP (73). Along these lines, Stability of Proteins from Rates of Oxidation (SPROX) (74) uses a chemical denaturation gradient and Solvent Proteome Profiling (SPP) (75) uses a solvent gradient instead of temperature. Finally, two additional approaches, termed Drug Affinity Responsive Target Stability (DARTS) (76) and Limited Proteolysis Mass Spectrometry (LiP-MS) (63) reveal information about protein binding through proteolytic footprints caused by metabolite binding.

Finally, like the many available tools that are available to predict well established PTMs, *in-silico* prediction of PTMs may be orthogonal to empirical methods. For example, lipid modification of proteins can be identified by using prediction models such as CSS-Palm 2.0 (77) or those based on the primary amino acid composition of k-spaced amino acid pairs (CKSAAP) (78). Furthermore, although recent breakthroughs in structure prediction using AlphaFold (79) did not encorporate ligand binding, emerging tools are undergoing preliminary work take ligands into account (80). These advances in identifying protein modification will be key in the discovery of metabolites that induce PTMs in cancer cells. How successful any of these small molecule-protein interaction profiling techniques (empirical or predicted) will be in determining protein interactors of newly discovered bioactive metabolites such as MMA, 3-IPA, PAHSAs, Phenyl-lactate, and others remains to be explored but could reveal valuable insight into the bioactivity of these metabolites. One difficulty in extending these methods from the study of exogenous small molecule engagement is the typically transient and low-affinity nature of metabolite-protein interactions. As such, we suggest that improved metabolite-protein interaction technologies will need to be developed to harness the therapeutic potential of bioactive metabolites.

## Conclusion

Bioactive, or “functional”, metabolites continue to emerge in human cells and tissues and may reveal previously underappreciated biological pathways in cancer while at the same time providing a scaffold from which new classes of small molecule therapeutics may be developed. Although we have briefly summarized several of the more exciting and recent findings in this regard, many other longstanding bioactive roles exist for metabolites that remain incompletely understood. Along these lines, new proteomics technologies have revealed underappreciated metabolite-protein interactions for well-known metabolites like ATP (63) for which the relevance is not yet known. The continued development of new technologies in metabolite-protein interactions and their application to understanding their bioactive roles will be an exciting and fruitful area of discovery moving forward.

## Author contributions

JM conceptualized, edited, prepared illustrations, and supervised the assembly of the manuscript. OP wrote and edited manuscript text. BB and MS edited the manuscript. All authors contributed to the article and approved the submitted version.

## Funding

We would like to acknowledge our funders. MS and BB receive funding from the MITACS Accelerate program. OP and JM receive funds from the Cancer Research Training Program of the Beatrice Hunter Cancer Research Institute, with funds provided by the Canadian Cancer Society’s JD Irving, Limited – Excellence in Cancer Research Fund.

## Conflict of interest

The authors declare that the research was conducted in the absence of any commercial or financial relationships that could be construed as a potential conflict of interest.

## Publisher’s note

All claims expressed in this article are solely those of the authors and do not necessarily represent those of their affiliated organizations, or those of the publisher, the editors and the reviewers. Any product that may be evaluated in this article, or claim that may be made by its manufacturer, is not guaranteed or endorsed by the publisher.
